# The proteomic effects of ketone bodies: implications for proteostasis and brain proteinopathies

**DOI:** 10.3389/fnmol.2023.1214092

**Published:** 2023-07-27

**Authors:** Lizbeth García-Velázquez, Lourdes Massieu

**Affiliations:** Department of Molecular Neuropathology, Instituto de Fisiología Celular, Universidad Nacional Autónoma de México (UNAM), México City, Mexico

**Keywords:** ketone bodies, proteome adaptation, β-hydroxybutyrate, β-hydroxybutyrylation, unfolded protein response, autophagy, brain proteinopathies

## Abstract

A growing body of evidence supports the beneficial effects of the ketone bodies (KBs), acetoacetate and β-hydroxybutyrate (BHB), on diverse physiological processes and diseases. Hence, KBs have been suggested as therapeutic tools for neurodegenerative diseases. KBs are an alternative fuel during fasting and starvation as they can be converted to Ac-CoA to produce ATP. A ketogenic diet (KD), enriched in fats and low in carbohydrates, induces KB production in the liver and favors their use in the brain. BHB is the most abundant KB in the circulation; in addition to its role as energy fuel, it exerts many actions that impact the set of proteins in the cell and tissue. BHB can covalently bind to proteins in lysine residues as a new post-translational modification (PTM) named β-hydroxybutyrylation (Kbhb). Kbhb has been identified in many proteins where Kbhb sites can be critical for binding to other proteins or cofactors. Kbhb is mostly found in proteins involved in chromatin structure, DNA repair, regulation of spliceosome, transcription, and oxidative phosphorylation. Histones are the most studied family of proteins with this PTM, and H3K9bhb is the best studied histone mark. Their target genes are mainly related to cell metabolism, chromatin remodeling and the control of circadian rhythms. The role of Kbhb on physiological processes is poorly known, but it might link KB metabolism to cell signaling and genome regulation. BHB also impacts the proteome by influencing proteostasis. This KB can modulate the Unfolded Protein Response (UPR) and autophagy, two processes involved in the maintenance of protein homeostasis through the clearance of accumulated unfolded and damaged proteins. BHB can support proteostasis and regulate the UPR to promote metabolism adaptation in the liver and prevent cell damage in the brain. Also, BHB stimulates autophagy aiding to the degradation of accumulated proteins. Protein aggregation is common to proteinopathies like Alzheimer’s (AD) and Parkinson’s (PD) diseases, where the KD and BHB treatment have shown favorable effects. In the present review, the current literature supporting the effects of KBs on proteome conformation and proteostasis is discussed, as well as its possible impact on AD and PD.

## 1. Introduction

Ketone bodies (KBs) have been widely acknowledged as alternative energy sources in conditions of low glucose availability. They are highly conserved across all life domains as they play a crucial role in helping organisms adapt metabolically to the presence or absence of nutrients. However, the role of KBs is not restricted to optimizing metabolism, they also link the external environment to changes in cellular function (Gut and Verdin, 2013; Newman and Verdin, 2017).

KBs include acetoacetate (AcAc), β-hydroxybutyrate (BHB), and acetone (Ac), from which BHB is the most abundant (Puchalska and Crawford, 2017). In humans, the plasma concentration of KBs fluctuates around 0.05–0.1 mM under physiological conditions. However, in response to glucose deprivation, starvation, a ketogenic diet, prolonged exercise, and insulin deficiency, its plasma concentration can reach 5–7 mM or even 20 mM, indicative of ketoacidosis (Gershuni et al., 2018).

BHB constitutes around 70% of the circulating KB pool and is used as an alternative source of energy to glucose in all extra-hepatic tissues. Remarkably, KBs are crucial for the brain during fasting periods as they can account for up to 75% of energy expenditure (Owen et al., 1967; Westman et al., 2007; Achanta and Rae, 2017).

The chemical nature of BHB not only confers this molecule with bioenergetic properties but also allows it to modify cellular signaling through the binding to G protein-coupled receptors (GPCRs) and inhibition of histone deacetylases HDACs (Taggart et al., 2005; Shimazu et al., 2013; Spigoni et al., 2022). Some other of its effects are particularly important for the brain, as accumulating evidence suggests that mild ketonemia can have neuroprotective effects by improving mitochondrial function, enhancing ATP production, and reducing inflammatory processes and oxidative stress (Shimazu et al., 2013; Youm et al., 2015; Li et al., 2021). Furthermore, KBs have shown neuroprotective effects in Alzheimer’s (AD) and Parkinson’s (PD) disease models (Kashiwaya et al., 2000).

BHB has a profound impact on the proteome by three different mechanisms. Firstly, BHB treatment can alter the proteome composition leading to the enrichment of specific pathways primarily related to metabolism (Düking et al., 2022; Zhu et al., 2023). Secondly, BHB covalently binds to proteins as a post-translational modification (PTM) known as β-hydroxybutyrylation, which occurs in numerous proteins, including histones (Xie et al., 2016). Thirdly, BHB regulates proteostasis by influencing two critical processes for maintaining protein quality control: the unfolded protein response (UPR) and autophagy (Finn and Dice, 2005; Guo et al., 2018).

Here, we summarize the current evidence on the impact of KBs, particularly BHB, on the proteome and its putative relevance for biological processes. We look into the mechanisms that drive changes in proteome composition, the molecules involved in the deposition and removal of β-hydroxybutyrylation, and the effects of BHB on the UPR and autophagy. Considering the significant relevance of BHB on the proteome, we will also discuss its effects on proteinopathies such as AD and PD, where interventions involving BHB have shown promising results.

## 2. Ketone bodies and their multifaceted roles

### 2.1. The synthesis process of KBs and its regulation

The synthesis of KBs, known as ketogenesis, takes place within the mitochondria of hepatocytes, and to a lesser extent in brain astrocytes, *Lgr5^+^* intestinal stem cells, and T-cells (Auestad et al., 1991; Achanta and Rae, 2017; Cheng et al., 2019; Zhang et al., 2020). Hepatocytes take up fatty acids from the bloodstream after lipolysis in adipocytes. Within the hepatocytes, acetyl-coenzyme-A acetyltransferase (ACAT) uses two molecules of acetyl-CoA to catalyze the formation of acetoacetyl-CoA (AcAc-CoA). Then, AcAc-CoA is converted to 3-hydroxymethylglutaryl-CoA (HMG-CoA) by the action of 3-hydroxy-3-methylglutaryl-CoA synthase 2 (HMGCS). HMG-CoA is cleaved by HMG-CoA lyase to produce acetyl-CoA and the ketone body, acetoacetate (AcAc). AcAc can be transported through the bloodstream to peripheral tissues or be a substrate for BHB or acetone production. AcAc can either be spontaneously decarboxylated to CO_2_ and acetone, which is highly volatile and exhaled through the lungs or be reduced to BHB by 3-hydroxybutyrate dehydrogenase 1 (BDH1). In peripheral tissues, BHB is oxidized into AcAc in the reverse reaction catalyzed by BDH1 (Newman and Verdin, 2017; Soto-Mota et al., 2020).

The rate of ketogenesis can be regulated at different levels. It is primarily dependent on the synthesis of HMG-CoA by HMGCS2. This process is regulated by the concentration ratios of acetyl-CoA to acetoacetyl-CoA and acetyl-CoA to free CoA-SH. Additionally, the availability of acetyl-CoA, derived either from fatty acid β-oxidation or from the catabolism of ketogenic amino acids, also influences ketogenesis. Notably, during the fed state the transport of fatty acids and their catabolism into ketone bodies is hampered (Huth et al., 1975; Grabacka et al., 2016).

At the transcriptional level, ketogenesis is regulated by many factors, with peroxisome proliferator-activated receptor alpha (PPARα) exerting a fundamental role as it orchestrates the transcription of genes involved in fatty acid transport, uptake and oxidation, as well as the synthesis and import of ketone bodies (Kersten et al., 2000; Pawlak et al., 2015). These include fatty acid binding protein (FABP), carnitine palmitoyltransferase 1A (CPT1A), peroxisomal acyl-CoA oxidase, mitochondrial long and medium chain acyl-CoA dehydrogenases (LCAD, MCAD), and the rate-limiting enzyme of ketogenesis, HMGCS2 (Meertens et al., 1998; Grabacka et al., 2016). However, it is important to note that the transcription of *HMGCS2* is influenced by multiple transcription factors including CREB, SP1, COUP-TF, DKHRL1, and FOXA2 (Yu and Reddy, 2007).

Most importantly, HMGCS2 activity is intricately linked to its PTMs, including palmitoylation, acetylation, and succinylation (Kostiuk et al., 2010; Grabacka et al., 2016). When palmitoylated, HMGCS2 binds PPARα and facilitates its own transcription (Kostiuk et al., 2010). Conversely, acetylation and succinylation lead to the inactivation of the enzyme and sirtuins can remove these PTMs. Particularly, during periods of fasting, the mitochondrial deacetylase, Sirt3, plays a crucial role in removing acetyl groups, thus activating HMGCS2 (Hirschey et al., 2010). On the other hand, succinylation has been identified in various proteins involved in both ketogenesis and fatty acid oxidation. Notably, HMGCS2 is the most heavily succinylated among these proteins, with 15 lysine residues serving as potential succinylation sites. The removal of this PTM is facilitated by the mitochondrial deacetylase/desuccinylase, Sirt5 (Rardin et al., 2013).

At the hormonal level, ketogenesis is strongly regulated by insulin, as it inhibits the breakdown of stored fats in adipocytes and promotes glucose uptake and utilization, leading to elevated levels of inhibitors of fatty acid oxidation and KBs production, such as succinyl-CoA and malonyl-CoA (Johnston and Alberti, 1982). Conversely, when insulin levels are low, catabolic hormones such as glucagon, cortisol, growth hormone, catecholamines (epinephrine and norepinephrine), and thyroid hormones stimulate lipolysis resulting in the release of free fatty acids from adipose tissue. These hormones also facilitate the transport of fatty acids to the liver and skeletal muscle. The increased influx of fatty acids into the liver promotes their β-oxidation and conversion to KBs (Bahnsen et al., 1984; Grabacka et al., 2016).

In summary, the synthesis of KBs is governed by a complex interplay of regulatory mechanisms that control their production in response to the physiological needs of the organism. For a more comprehensive exploration of the regulatory aspects of ketogenesis, see Grabacka et al. (2016). It is worth noting that alterations in KBs synthesis and metabolism may influence the impact of BHB on the proteome, given the dose-dependent nature of processes such as protein β-hydroxybutyrylation.

### 2.2. The role of KBs beyond energy fuels

In the last decades, the function of classical metabolites like lactate, succinate, α-ketoglutarate, butyrate, propionate, acetate and now KBs, has been reconsidered, as many of them, either produced by the eukaryote or its microbiota, can activate signaling pathways that impact the cellular physiology (Blad et al., 2012; Canfora et al., 2015; Chriett and Pirola, 2015; Kasubuchi et al., 2015; Newman and Verdin, 2017). Furthermore, they promote PTMs that alter the proteome in response to environmental conditions (Xu et al., 2022).

The cellular effects of AcAc remain poorly explored due to its chemical instability. Nevertheless, studies have shown that it promotes the secretion of interleukin (IL)-6, up-regulates intercellular adhesion molecule 1 (ICAM1), induces insulin release *in vitro* and decreases the generation of free oxygen radicals (Hoffman et al., 2002; Jain et al., 2003; Abdelmegeed et al., 2004; Noh et al., 2006; Haces et al., 2008; MacDonald et al., 2008). It also binds to the G-protein coupled receptor GPR43 to increase plasma lipid utilization by controlling the activation of lipoprotein lipase (LPL) via adipose GPR43 (Miyamoto et al., 2019; Spigoni et al., 2022). Decreased production of reactive oxygen species and increased ATP synthesis by acetoacetate, have been mainly associated to its protective action against glutamate toxicity in cultured neurons (Noh et al., 2006; Haces et al., 2008).

On the other hand, BHB is a small polar molecule that can be found as R/D-BHB or L/S-BHB enantiomers due to its chirality at the 3′ hydroxyl group. However, the S-BHB is not a product of human or mouse metabolism. Due to enzymatic specificity, only R-BHB can be synthetized and metabolized into acetyl-CoA and ATP (Newman and Verdin, 2017). BHB is more stable and abundant than AcAc and acetone and it has even been found in a polymerized form in intracellular granules in the cytoplasm, where it acts to regulate intracellular signaling, mitochondrial function and calcium channel activity (Elustondo et al., 2013; Smithen et al., 2013; Dedkova and Blatter, 2014). Yet, BHB is found and acts mainly as a monomer in healthy cells (Dedkova and Blatter, 2014).

Besides its role as an energy metabolite, BHB is related to many other cellular functions and acts as a signaling molecule, as it binds to GPR41/FFAR3 and GPR109/HCAR2 (Taggart et al., 2005; Spigoni et al., 2022). The binding of BHB to GPR109 reduces atherosclerosis and inflammation (Taggart et al., 2005; Zhang et al., 2021b), while binding to GPR41 modulates lipid metabolism (Won et al., 2013; Miyamoto et al., 2019). It also regulates inflammation suppressing the activation of the nod-like receptor pyrin domain containing protein 3 (NLRP3) inflammasome and the subsequent caspase-1 activation and interleukin 1β (IL-1β) secretion (Youm et al., 2015).

Some BHB actions are important for the brain, especially under pathological conditions, as it enhances mitochondrial respiratory chain complex I activity, reduces oxidative stress, inhibits mitochondrial apoptosis, controls inflammation and stimulates the autophagic flux. Furthermore, it has been shown that BHB administration improves the neurological score and reduces the infarct volume after stroke (Rahman et al., 2014; Yin et al., 2015; Guo et al., 2018; Li et al., 2021; Montiel et al., 2023). However, the contribution of a specific mechanism to the protective effect of BHB against a particular pathologic state has not been completely elucidated.

In addition, BHB can influence the chromatin conformation by inhibiting HDACs and inducing histone β-hydroxybutyrylation (Shimazu et al., 2013; Xie et al., 2016). These two functions will be further described in the following sections for a more comprehensive understanding.

β-Hydroxybutyrate production has also an effect on feeding behavioral patterns such as food anticipation activity (FAA). It has been shown that the circadian clock gene, *Per2,* upregulates BHB synthesis enzymes and FAA, which is abated when the monocarboxylate transporter 1 (MCT1 and SLC16a1), crucial for BHB efflux, is deleted in the liver (Chavan et al., 2016; Martini et al., 2021). Moreover, the intracarotid infusion of KBs stimulates food intake mediated by the increased expression of orexigenic hypothalamic peptides. This effect has important implications for the development of obesity (Carneiro et al., 2016). In agreement, MCT1-deficient mice exhibit resistance to diet-induced obesity (Lengacher et al., 2013). These observations underline the need for comprehensive research on the physiologic and behavioral implications that may arise from prolonged exposure to ketosis.

On the other hand, it has been suggested that the circadian clock can regulate the diet-induced ketogenesis through the rhythmic activity of PPARα in the liver (Mezhnina et al., 2022), and conversely, KBs also influence the regulation of circadian processes (see Masi et al., 2022).

It is essential to consider that there is limited understanding of potential long-term adverse effects of increased BHB levels due to the limited follow-up duration of most of human studies. Diet-based approaches and exogenous supplementation of ketones need strict control of BHB blood levels, precluding its elevation beyond safe levels. Ketone therapy recommends moderate ketosis in the range of > 0.2 up to 5-8 mM (Veech, 2004; Hashim and VanItallie, 2014; Koppel and Swerdlow, 2018; Camberos-Luna and Massieu, 2020), which is considered safe and below the levels observed in diabetic ketoacidosis (> 0.8 up to 25 mM), which can cause cerebral edema and even the death of the patient (Bohn and Daneman, 2002). There are other limitations of the KD, remarkably the poor adherence of the patients due to its low palatability. In the long-term KD causes secondary effects, such as a loss of weight, especially relevant in aged patients, and increased blood levels of LDL cholesterol (Fuehrlein et al., 2004 Choi et al., 2020; Kolb et al., 2021). Also, prolonged treatments with sodium ketone salts cause gastrointestinal distress, cation overload, and acidosis (Choi et al., 2020; Kolb et al., 2021). Similarly, ketosis induction by the intake of medium chain triglycerides causes gastrointestinal complications such as diarrhea, dyspepsia, and flatulence (Thorburn et al., 2006; Wang et al., 2021).

Therefore, it is essential to conduct further research on the long-term effects of KB in order to estimate their adverse effects and establish the most adequate time of onset and treatment duration.

Other effects of KB include an initial moderate increase in the production of reactive oxygen species after mitochondrial ketolysis and the production of inflammatory cytokines. However, this might be an adaptive defense response as it increases the tissue resistance to subsequent insults, such as ischemia, due to the induction of Nfr2, a master regulator of antioxidant response genes (Al-Zaid et al., 2007; Shi et al., 2014; Kanikarla-Marie and Jain, 2015). These observations suggest that a period of adaptation is necessary before the beneficial effects of KBs are evident. Hence, attention should be paid to the period when KBs effects are evaluated.

## 3. Ketone bodies induce changes in the proteome pattern

The rise of the physiological levels of ketone bodies whether induced by KD, starvation or the administration of BHB or its derivatives, has an impact in genome regulation and therefore in the protein content (Xie et al., 2016; Cornuti et al., 2023).

The observed changes in the genome regulation are achieved mainly by two mechanisms attributable to BHB. One notable mechanism involves the induction of lysine β-hydroxybutyrylation (Kbhb) of histones, which is related to a transcriptional active state of the chromatin and will be further detailed in the following section. On the other hand, BHB was originally identified as an endogenous inhibitor of class I and II-a HDAC (Shimazu et al., 2013).

Histone hyperacetylation is generally considered an active gene expression mark; thereby, HDAC induces a transcriptionally repressive state. *In vitro* studies using purified HDAC proteins have demonstrated that BHB has a direct inhibitory effect on HDAC function, which may be due to its structural similarity to the HDAC inhibitor butyrate (Shimazu et al., 2013; Newman and Verdin, 2017). The inhibitory effect of BHB on HDAC occurs particularly on lysines 9 and 14 of histone 3 (H3K9/K14) in HEK293T cells, in which the acetylation shows a dose-dependent effect (Shimazu et al., 2013). A similar outcome has been observed in H3 acetylation levels in primary cortical neurons and mouse bone marrow-derived macrophages exposed to D-BHB (Youm et al., 2015; Sleiman et al., 2016). Furthermore, the inhibitory effect of HDAC by BHB was also observed *in vivo*; mice with high BHB levels induced by fasting, caloric restriction, or the administration of BHB through a subcutaneous pump had increased levels of H3K9ac and H3K14ac in the kidney (Shimazu et al., 2013).

However, controversy has emerged about the inhibitory effect of BHB on HDACs, since the exposure of cell cultures, including HEK293, HMEC-1, rat L6 myotubes, and human primary myotubes to sodium D-BHB had no effect on H3 acetylation even at concentrations up to 40 mM (Chriett et al., 2019). This finding is consistent with the study by Chen et al. (2017), which showed that increasing concentrations of BHB did not show a statistically significant increase in the H3K9ac mark in cortical neurons (Chen et al., 2017). Furthermore, when HEK293 cells were treated with up to 20 mM Na-BHB, only marginal increases in histone acetylation were detected. A similar outcome was observed in the liver of 48 h fasted or streptozotocin-treated mice (Xie et al., 2016).

These controversial results highlight the relevance of further research on this potential effect using diverse cell types, physiological contexts, and experimental approaches, in order to understand whether the observed effects are directly attributable to BHB, its metabolism intermediaries or are the result of contextual factors. Moreover, in most of these studies, very high concentrations of BHB (10–20 mM) were used, which are far from the physiological concentrations of KBs and more likely correspond to ketonemia. Thus, more studies are needed to assess the specificity of this effect in physiological conditions.

Regardless of the underlying mechanism, KBs induce alterations in the genome and the transcriptome that ultimately reflect changes in the proteome composition (Table 1). The extent of these changes is influenced by various factors such as cell type and developmental stage, among others. In the mouse hippocampal neuronal cell line HT22, BHB induces the upregulation of proteins related to acetylation and methylation processes. Further analysis in this study showed that BHB has neuroprotective effects by influencing chromatin bivalency (Zhu et al., 2023). In this study, a 0.2 mM concentration of BHB was used, suggesting that at physiological levels, BHB can induce protein acetylation and methylation.

**Table 1:** Protein pathway enrichment in response to ketone bodies (KB).

Cell type/tissue	Treatment	Main pathways enriched	Reference
HT22 cells	BHB 0.2 mM	Upregulation: Protein acetylation and processes related to protein methylation.	Zhu et al. (2023)
Mouse cortical neurons	Ketogenic diet	Upregulation: Synaptic cycle, OXPHOS, glycolysis, TCA, AA metabolism, ketolysis and endo/lysosome processing.	Düking et al. (2022)
Mouse cortical astrocytes		Upregulation: β-oxidation, AA breakdown, OXPHOS and ketolysis. Downregulation: PPP, proteasome, AA metabolism and glycolysis.	
Mouse cortical microglia		Upregulation: Proteasome processing. Downregulation: OXPHOS, AA breakdown, TCA and β-oxidation.	
Mouse cortical endothelial cells		Downregulation: AA breakdown and TCA.	
Mouse cortical oligodendrocytes		Upregulation: Ion/Vesicular transport.	

Summary of the functions of the proteins up- or down-regulated in response to BHB related treatments in CNS cells.

BHB, β-Hydroxybutyrate. OXPHOS, Oxidative phosphorylation system. TCA, Tricarboxylic acid cycle. AA, Amino acid. PPP, Pentose phosphate pathway.

Düking et al. (2022), investigated how the brain adapts its metabolism in response to limited glucose availability by feeding mice with a KD at different postweaning ages. A proteome atlas of the major cell types in the central nervous system (CNS) was generated. The findings revealed that each of the CNS cell types employed distinct strategies to cope with the altered availability of energy metabolites. Specifically, astrocytes and neurons exhibited the major metabolic plasticity, while oligodendrocytes demonstrated less adaptability (Düking et al., 2022; Table 1). Interestingly, in this study the long-term effects of a KD were investigated. The density of astrocytes, neurons, oligodendrocytes and microglia was analyzed in brain after feeding a KD for 180 days. Since mice did not show changes in the density of any cell type, the authors suggested that the KD caused no brain damage. However, markers of cellular stress or cell death were not evaluated.

The aforementioned studies highlight the capacity of cells to adapt their proteome composition in response to elevated KBs. This phenomenon is especially relevant in the brain, which heavily relies on KBs when glucose is scarce. Interestingly, despite facing similar conditions, cells employ diverse strategies to maintain their homeostasis.

### 3.1. β-Hydroxybutyrate binds to proteins as a post-translational modification

#### 3.1.1. Lysine β-hydroxybutyrylation (Kbhb)

The unique chemical and physical properties of β-hydroxybutyrate (BHB) enable this monocarboxylate to form covalent bonds with lysine residues, resulting in a novel post-translational modification known as β-hydroxybutyrylation (Kbhb). Kbhb is a well conserved PTM, found in all the species analyzed, including the *Homo sapiens*, *Mus musculus*, *Rattus norvegicus*, *Saccharomyces cerevisiae* and *Drosophila melanogaster* (Xie et al., 2016; Luo et al., 2023). The abundance of this mark changes in response to BHB levels in a dose-dependent manner (Xie et al., 2016).

Histones were the first protein family identified with Kbhb sites (Xie et al., 2016). To date, Kbhb is well recognized as a widespread histone mark and comprehends 46 Kbhb sites distributed in H1.0, H2A type A, H2A type B, H2B, H3.1 and H4 proteins (Xie et al., 2016; Huang et al., 2021). This histone PTM has been observed *in vitro* and *in vivo*, and is related to an active transcriptional state, which is independent of histone acetylation (Xie et al., 2016). The β-hydroxybutyrylation of lysine 9 in histone 3 (H3K9bhb) is the most extensively studied Kbhb mark, known to be highly responsive to changes in BHB levels (Terranova et al., 2021). It is enriched in response to high KBs levels in the liver, kidney, brain, heart and the cell lines HEK293T and MEF (Xie et al., 2016; Huang et al., 2021; Koronowski et al., 2021; Hou et al., 2022; Cornuti et al., 2023; Luo et al., 2023). H3K9bhb has been related to the upregulation of a vast set of genes specific of each cell type or tissue.

The deposition of the H3K9bhb mark is influenced by the activity of two essential enzymes of the KBs metabolism, HMGCS2 and BDH1. The knockout of the *Hmgcs2* gene in small intestine crypt cells is related to a marked loss of H3K9bhb across thousands of promoters, affecting metabolic gene programs (Terranova et al., 2021). On the other hand, there is a negative correlation between the levels of BDH1 and the enrichment of the H3K9bhb mark in the hepatoma cell line PLC/PRF/5, where lower levels of BDH1 led to a higher expression of a variety of genes, including some related to a poor prognosis for hepatocellular carcinoma (Zhang et al., 2021a). Therefore, the enrichment of the H3K9bhb mark heavily depends on KBs production and has an impact on gene expression.

Changes in H3K9bhb enrichment are relevant in the brain because it can regulate genes involved in different processes crucial for brain function. When endogenous ketosis is induced in mice by 2-days starvation after post-weaning, H3K9bhb is increased in the cerebral cortex, where it changes the expression of genes related to circadian rhythms, neurogenesis, dendrite morphogenesis, chromatin remodeling, and synaptic transmission, among others. Furthermore, the changes in clock-core genes are correlated with altered locomotor circadian rhythmicity (Cornuti et al., 2023), suggesting that fasting can alter circadian rhythmicity of locomotor activity.

Apparently, all studies on H3K9bhb have used the PTM-1250 antibody, as it is the only commercially available antibody against H3K9bhb. However, a recent study raised concerns about the data obtained with this antibody, since it has a certain level of non-specificity as it also recognizes H3K9ac. Therefore, researchers should be cautious and consider the potential detection of alternative PTMs, such as H3K9ac or others, when using this antibody (Tsusaka et al., 2023). Nonetheless, studies have reported changes only H3K9bhb and not in H3K9ac in response to BHB (Xie et al., 2016). This suggests that despite its potential non-specificity, data obtained with the PTM-1250 may indeed reflect authentic alterations in H3K9bhb, or involve other unidentified PTM targeted by this antibody. Therefore, the development and of novel highly specific antibodies capable of distinguishing between different post-translational modifications (PTMs) involving acyl groups, is still needed.

Besides histones, p53 was one of the first β-hydroxybutyrylated proteins identified (Liu et al., 2019a,b). This protein regulates the expression of genes and microRNAs involved in cellular processes such as cell growth arrest, apoptosis, autophagy, ferroptosis, senescence, aging, oxidative balance and metabolism, and its activity is finely tuned by PTMs (Lozano, 2016; Liu et al., 2019a). Now β-hydroxybutyrylation is added to the list of p53 PTMs, since lysines 120, 319, and 370 are Kbhb sites. This modification is catalyzed by CBP/p300 and has functional effects, since it attenuates p53 acetylation and downregulates the transcriptional activity of target genes including *Cdkn1a (p21) Puma*, which are involved in cell cycle arrest and apoptosis, respectively (Liu et al., 2019b).

Proteomic studies have thus far identified over 3,000 Kbhb sites in at least 1,400 proteins in cell lines exposed to BHB and in rodent models subjected to treatments that promote endogenous ketosis such as fasting or feeding a high fat diet (Table 2). The identity and number of β-hydroxybutyrylated proteins vary depending on the cell type and physiological context, highlighting the specific and dynamic nature of this post-translational modification (PTM). As seen in Table 2, despite the diverse functions of the Kbhb proteins, there is an enrichment of β-hydroxybutyrylated proteins related to the regulation of the genome and its expression, including chromatin structure, transcript processing and DNA repair (Xie et al., 2016; Huang et al., 2021; Cornuti et al., 2023) Likewise, Kbhb is enriched in proteins related to fatty acid, amino acid and pyruvate metabolism, the regulation of tricarboxylic acid cycle, oxidative phosphorylation, and detoxification pathways, among others (Koronowski et al., 2021; Hou et al., 2022; Luo et al., 2023). Interestingly, 2-day starvation induced an increase in Kbhb in the cerebral cortex, mainly in proteins involved in transcription regulation, transcription dependent on RNA pol II, and morphogenesis (Cornuti et al., 2023).

**Table 2:** Identified β-hydroxybutyrylated proteins and their associated pathways.

Cell type/tissue	Treatment	Kbhb sites and proteins	Main enriched biological pathways	Reference
HEK 293 T cells	10 mM BHB for 24 h.	3,392 Kbhb sites in 1431 proteins.	DNA repair, spliceosome, ribosome, RNA transport and chromatin related proteins.	Huang et al. (2021)
MEF cells	5 mM BHB for 24 h.	840 unique Kbhb sites in 429 proteins.	Aminoacyl-tRNA biosynthesis, citrate cycle, metabolism of 2-oxocarboxylic acid, pyruvate, fructose, and mannose.	Hou et al. (2022)
Mouse liver	48 h fasting.	891 Kbhb sites in 267 proteins.	Fatty acid, amino acid, detoxification, and one-carbon metabolic pathways.	Koronowski et al. (2021)
Rat heart (diabetic cardiomyopathy model)	High fat and sugar diet + Streptozotocin.	3,520 kbhb sites in 1089 proteins.	TCA, OXPHOS, and propanoate metabolism.	Luo et al. (2023)
Mouse occipital cortex	48 h fasting.	234 β-hydroxybutyrilated proteins.	Regulation of transcription, regulation of transcription-dependent on RNA pol II and morphogenesis.	Cornuti et al. (2023)

The vast number of Kbhb residues and β-hydroxybutyrylated proteins that result from KB-related treatments is involved in a variety of biological processes in the different cell types and tissues.

BHB, β-Hydroxybutyrate. OXPHOS, Oxidative phosphorylation system. TCA, Tricarboxylic acid cycle.

Surprisingly, while approximately 53% of β-hydroxybutyrylated proteins contain only one Kbhb site, others harbor multiple or even more than ten Kbhb sites, as demonstrated in HEK293T cells treated with 10 mM BHB (Huang et al., 2021). In this same study, a protein complex enrichment analysis revealed that certain protein complexes exhibit β-hydroxybutyrylation in more than one protein, like the ubiquitin E3 ligase, the methyl-CpG–binding domain protein 1 (MeCP1) and the BRG-/BRM-associated factor (BAF) complexes (Huang et al., 2021). Four of the five subunits of the ubiquitin E3 ligase complex, eight of the nine subunits in the MeCP1 complex and six of the nine subunits in the BAF complex were β-hydroxybutyrylated, actually, the protein GATAD2B from the BAF complex contains 11 Kbhb sites (Huang et al., 2021).

Other proteins that have been found heavily modified by Kbhb are myosin heavy chain 9 (Myh9) with 17 sites, heat shock protein HSP 90-β (HSP90ab1) with 16 sites and plectin (Plec) with 13 sites (Hou et al., 2022). It is noteworthy, that some of the Kbhb are located in regions critical for the binding of enzymatic cofactors or co-enzymes, or in sites that alter the protein function when mutated (Huang et al., 2021). Therefore, there is a possibility that high levels of Kbhb might disrupt the normal activity of the target proteins and that β-hydroxybutyrylation is an inducible PTM that can affect protein function. Furthermore, there may be a coordinated regulation of this modification within specific protein complexes enriched with this mark.

**Figure 1:**
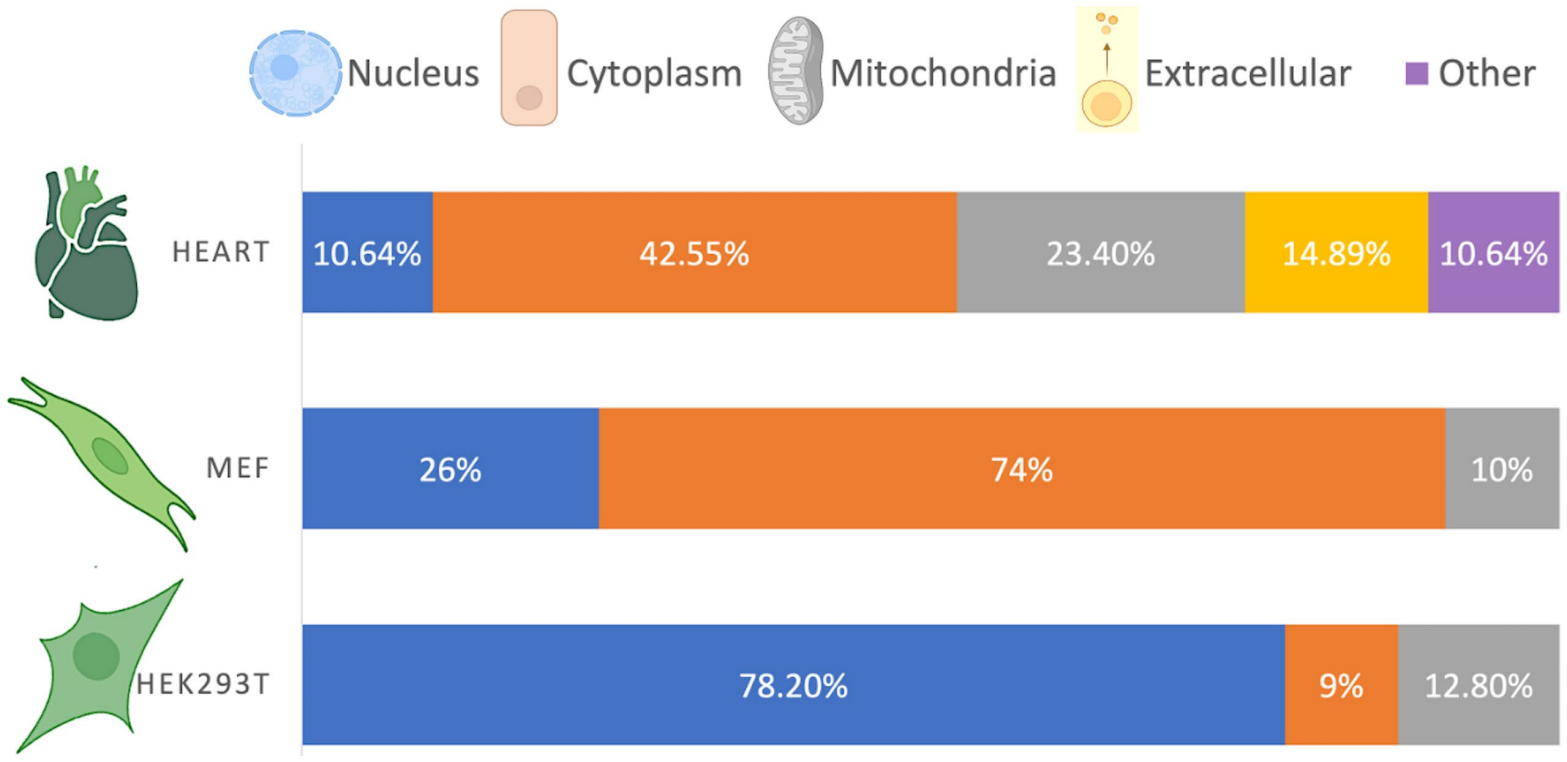
Cellular distribution of β-hydroxybutyrylated proteins. The cellular compartmentalization of Kbhb proteins varies among different cell types, exhibiting a non-homogeneous distribution. Based on Luo et al. (2023), Huang et al. (2021), and Hou et al. (2022).

The cellular compartmentalization of β-hydroxybutyrylated proteins varies according to cell type or tissue (Figure 1). In the heart of diabetic rats, β-hydroxybutyrylated proteins are more abundant in the cytoplasm, followed by the mitochondria, nucleus and extracellular space (Luo et al., 2023). However, in HEK293T cells, proteins with Kbhb sites are more abundant in the nucleus, followed by the mitochondria, while in MEF cells most of the Kbhb proteins were located in the cytosol (Huang et al., 2021; Hou et al., 2022).

Although the distribution of β-hydroxybutyrylated proteins differs depending on the cell type or tissue, it is in accordance with the abundance and function of the enriched pathways. For instance, the higher abundance of Kbhb proteins in the nucleus of HEK293T cells is consistent with their involvement in DNA repair, spliceosomes, ribosomes, and RNA transport, while the higher abundance of Kbhb proteins in the cytosol of MEF cells is consistent with their function in cellular metabolism (Huang et al., 2021). The compartmentalization of β-hydroxybutyrylated proteins in cells from the nervous system has not been yet reported.

Further investigations are warranted to expand the analysis to a wider range of cell types and tissues. Moreover, it is important to support these observations using imaging techniques, as the current data are based solely on predictions derived from sequence analysis.

It is noteworthy that most of the studies on Kbhb have used concentrations from 5 to 10 mM of BHB, which corresponds to the concentrations reached during ketosis. Hence, the information provided by these studies would be more related to proteome modifications in ketonemia rather than physiological conditions. Interestingly, in the cerebral cortex, some proteins have been found to be β-hydroxybutyrylated in mice maintained under *ad libitum* feeding conditions, including those related to nervous system development. This observation suggests that even in control feeding conditions some proteins contain this PTM. Therefore, more investigation is needed in order to elucidate the implications of basal levels of Kbhb on physiological processes and its changes in pathological states.

#### 3.1.2. Kbhb: molecular players in sight

Efforts have been made to gain a deeper understanding of β-hydroxybutyrylation by identifying two key elements: consensus sequences that are more likely to be β-hydroxybutyrylated and the machinery responsible for the Kbhb modification establishment and removal. Although no single consensus sequence has been identified to date, two distinct patterns have been observed. In MEF cells, an enriched motif for Kbhb is characterized by an overrepresentation of hydrophobic amino acids, Leu and Phe at the +1 position, while acidic amino acids Asp or Glu are enriched at the + 3 and − 1 positions (Hou et al., 2022). In contrast, in HEK293T cells, positively charged lysine is favored at most positions (−6, −5, −4, −3, +3, +4, +5, and + 6), while negatively charged amino acids are underrepresented at the −2 position (Huang et al., 2021). These findings suggest that the establishment of β-hydroxybutyrylation may not be dictated by a consensus sequence, but rather influenced by the cellular context. However, additional investigations are required to confirm this hypothesis.

**Figure 2:**
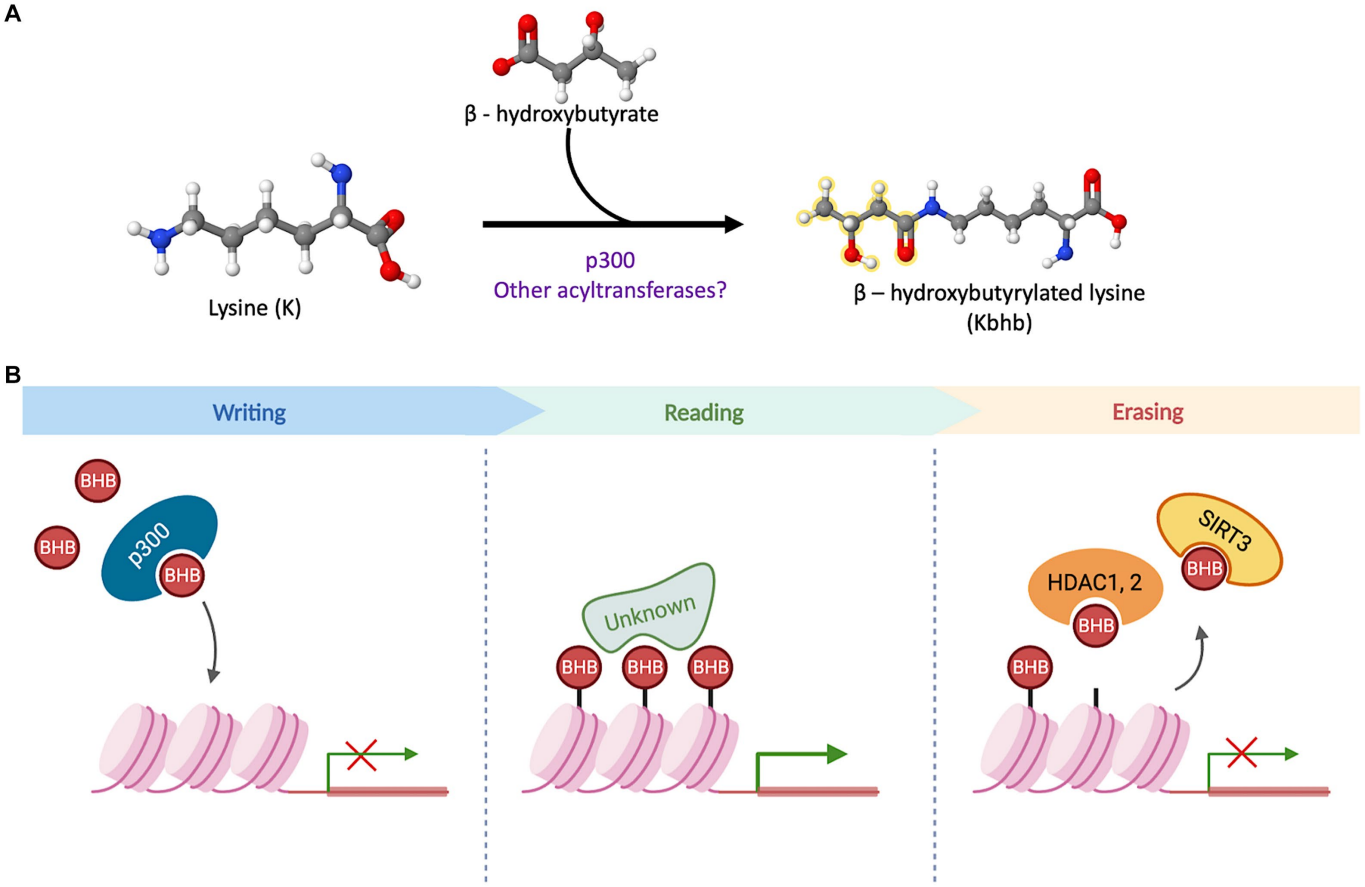
β-hydroxybutyrylation and its molecular players. **(A)** Schematic representation of the binding of D-BHB to a Lys residue to form Kbhb. The reaction can be favored by p300 or other undiscovered acyltransferases. **(B)** The acyltransferase p300 has a binding pocket capable of accommodating and transferring uncharged short-chain acyl-CoAs. In a cellular context, it acts as a histone BHB transferase that promotes the transcriptional activity of target genes. The proteins with a “reader” function for Kbhb have not been identified to date. In cell culture models, this PTM can be removed by the sirtuin 3 (Sirt3) and the histone deacetylases HDAC1 and 2.

The quest to unravel the molecular mechanisms underlying β-hydroxybutyrylation has successfully identified “writer” and “eraser” proteins (Figure 2). However, the search for “reader” proteins that can interpret or decode this modification is still ongoing, and their identification will be crucial in comprehending the processes and outcomes associated with this PTM.

The search for writer Kbhb proteins, led to the identification of p300, a well-known acyltransferase. The p300 protein possesses a binding pocket capable of accommodating and transferring uncharged short-chain acyl-CoAs, enabling acetylation, propionylation, and crotonylation of lysines (Dancy and Cole, 2015; Kaczmarska et al., 2017). *In vitro* assays involving the addition of p300 and bhb-CoA demonstrated an increase in Kbhb sites on core histones, indicating that p300 functions as a histone Kbhb transferase on nucleosomes and can directly modulate transcription (Huang et al., 2021). Moreover, a knockout model of p300 in HCT116 cells exhibited decreased levels of multiple histone Kbhb sites, while p300 pharmacological inhibition led to reduced levels of both Kac (lysine acetylation) and Kbhb on H3K9, H3K18 and H4K8 sites in a dose-dependent manner (Huang et al., 2021). In contrast, when p300 or CREB binding protein (CBP) were overexpressed, it resulted in an increase in H3K18bhb and H4K8bhb levels. However, this effect was not observed when other acyl-transferases, such as GCN5 or PCAF, were overexpressed (Huang et al., 2021). These findings suggest that p300 plays a crucial role in regulating histone Kbhb levels. Moreover, these results highlight the distinct functions of various acyl-transferases in the context of β-hydroxybutyrylation, further emphasizing the complexity and specificity of this epigenetic modification.

Regarding the removal of Kbhb mark, it was demonstrated that Sirt3 acts as an eraser protein for H3K9bhb in HEK293T cells and in cell-free assays. Sirt3 exhibits class-selective histone de-β-hydroxybutyrylase activities, with a preference for removing Kbhb from H3 K4, K9, K18, K23 and K27, while, Sirt4 and Sirt7 do not display any enzymatic activity towards H3K9bhb removal (Zhang et al., 2019). Sirt3 is normally located in mitochondria where it is mainly involved in the deacetylation of mitochondrial proteins, however, it was also found in the nucleus in HEK293T (Zhang et al., 2019), where it acts as a histone Kbhb eraser. Furthermore, histone deacetylases HDAC1 to HDAC3, as well as Sirt1 and Sirt2, have been identified as eraser Kbhb proteins in cell-free assays. However, in HEK293T and HeLa cells, HDAC1 and HDAC2 were found to exhibit the primary Kbhb deacylase activity, for which they may have overlapping removal functions (Huang et al., 2021).

In summary, identifying proteins involved in β-hydroxybutyrylation establishment and removal provides important insights into the molecular regulation of this modification. However, these findings also emphasize the need to continue the search for additional molecules involved in β-hydroxybutyrylation regulation in order to fully comprehend the contextual factors influencing its establishment, removal, and outcomes.

## 4. β-Hydroxybutyrate versus a dysregulated proteostasis

### 4.1. Ketone bodies attenuate the unfolded protein response

To ensure protein folding fidelity and maintain endoplasmic reticulum (ER) functions, the unfolded protein response (UPR) evolved to a network of signal transduction pathways in eukaryotic cells, to reprogram gene transcription, mRNA translation and protein modifications, and relieve the load of unfolded or misfolded proteins in the ER to restore protein homeostasis (Hetz et al., 2020).

The UPR is triggered by ER stress whenever misfolded or unfolded proteins accumulate in the ER lumen. Three transmembrane sensors residing in the ER membrane dissociate from the chaperone protein BIP/GRP78 and activate the three branches of the UPR; the PKR-like endoplasmic reticulum kinase (PERK), the inositol requiring enzyme-1α (IRE1α), which shows both kinase and RNase activities, and the activating transcription factor 6 (ATF-6), to reestablish proteostasis. The activation of PERK leads to the transient inhibition of cap-dependent protein synthesis to reduce the protein load in the ER, through the phosphorylation of the eukaryotic translation initiator factor (eIF2α), and the selective translation of activating transcription factor 4 (ATF4) mRNA. ATF4 increases the expression of genes involved in ER protein folding and degradation, autophagy, amino acid transport and redox regulation. However, under prolonged PERK activation, apoptosis can be triggered through the induction of the transcription factor CHOP/GADD153, leading to the upregulation of apoptotic genes and the downregulation of anti-apoptotic molecules (Malhotra and Kaufman, 2007; Merksamer and Papa, 2010). IRE1α pathway is conserved from yeast to mammals and the activation of its RNase activity leads to the splicing of a 26 nucleotides sequence of the X-box-binding protein 1 (XBP1) mRNA, leading to a shift in the open reading frame, which drives the production of a larger protein with transcription factor activity. Spliced XBP1 (XBP1s) upregulates the synthesis of chaperons and proteins of the ER-associated degradation (ERAD). Finally, dissociation of ATF6 from BIP/GRP78 leads to its transit to the Golgi apparatus where it is cleaved, allowing its translocation to the nucleus to enhance the expression of chaperons and ERAD components (Malhotra and Kaufman, 2007; Merksamer and Papa, 2010).

Failure or sustained activation of the UPR may lead the cell onto apoptotic pathways that eventually result in autophagy or cell death (Tagawa et al., 2019). However, the UPR can also be involved in other processes as metabolism regulation. Its activation can mediate metabolic changes in the liver and conversely, the UPR can also be activated by a metabolic shift in the liver (Ma et al., 2021). Furthermore, impairment of the UPR has been implicated in metabolic dysregulation (Shao et al., 2014).

Mice treated with tunicamycin, which is an ER stressor, exhibited alterations in their liver transcriptome. There was an increase in the expression of genes involved in monocarboxylic acid metabolism, lipid synthesis and catabolism, fatty acid metabolism and genes of enzymes involved in the synthesis and degradation of KBs, including HMGCS and BHD. In agreement with transcriptome data, metabolomic analysis revealed the enrichment of metabolites involved in mitochondrial β-oxidation and KBs metabolism (Ma et al., 2021). These data suggest that KBs metabolism can be stimulated under ER stress conditions and UPR activation in the liver.

A metabolic shift induced by fasting or the KD triggers the UPR activation in the liver by activating the IRE1α-XBP1 branch. Hepatocyte-specific deletion of IRE1α abrogates IRE1α-XBP1 activation and disrupts fatty acid oxidation and ketogenesis, suggesting the failure of the adaptive metabolic shift to fasting. PPARα, the master regulator of the starvation response, is regulated by XBP1s binding to its promoter. IRE1α deleted mice failed to upregulate PPARα and its target genes involved in fatty acid oxidation and ketogenesis, further suggesting a crucial role of IRE1α-XBP1 in liver metabolic adaptation to fasting. Altogether, these data support that ketosis induction by fasting or the KD activates IRE1α RNase activity producing XBP1s, which upregulates ketogenesis allowing a metabolic adaptation in the liver (Shao et al., 2014). However, it is unknown whether XBP1 splicing and PPARα upregulation are related to Kbhb.

Metabolic regulation by the UPR has not been investigated in brain. A recent study revealed that 90-day feeding a KD in mice induces opposite changes in the UPR pathway in astrocytes and neurons. While this pathway was upregulated in neurons, downregulation was observed in astrocytes (Koppel et al., 2021). Based on these data, it can be suggested that the UPR-mediated metabolic adaptation to ketosis might be differentially regulated in astrocytes and neurons.

In the context of aging, 3-days starvation attenuates the UPR and the activation of the NLRP3 inflammasome in the liver of aged rats. Also, BHB treatment in HepG2 human hepatocyte cell line, inhibited UPR and NLRP3 inflammasome activation induced by palmitate. This effect is associated with increased antioxidant proteins through the upregulation of FOXO3a and decreased reactive oxygen species production. These results suggest that BHB treatment can counteract the aged-associated UPR activation and protect the liver against age-induced oxidative stress and inflammation (Bae et al., 2016). Similarly, Tagawa et al. (2019) reported in hepatoma cells, that BHB exposure suppresses tunicamycin-induced ER stress and increases cell viability through apoptosis inhibition (Tagawa et al., 2019). BHB intraperitoneal treatment also reduced the abundance of UPR proteins in the liver. These results differ from those of the study by Shao et al. (2014), where starvation and the KD activated the IRE1α branch of the UPR to drive a metabolic shift in the liver toward ketogenesis (Shao et al., 2014). This discrepancy suggests that the effects of ketosis and BHB administration are context dependent, and might differ under normal conditions or ER stress.

In agreement with the aforementioned results, it was reported that 3 weeks feeding a KD previous to brain ischemia in mice, attenuated the ischemia-induced activation of the PERK branch of the UPR and the NLRP3 inflammasome, further suggesting that BHB can downregulate ER stress in the context of ischemia in the brain (Guo et al., 2018). A recent study reported that the exogenous administration of BHB post-ischemia downregulates both the PERK and the IRE1α branches of the UPR (Montiel et al., 2023).

BHB also protects against ER stress-associated vascular endothelial cell damage under low glucose. Exposure of human umbilical vein endothelial cells (HUVECs) to low-glucose for 24 or 48 h, induced ER stress responses mediated by the increased phosphorylation of PERK and the cleavage of activating transcription factor 6 (ATF6), while the splicing of Xbp1 mRNA decreased. The increase in PERK pathway proteins induced by glucose deficiency was attenuated by supplementation with BHB, which also protected from vascular endothelial cell damage (Soejima et al., 2018), supporting a protective role of BHB against ER stress activated under energy limiting conditions.

BHB can activate G-protein coupled transmembrane receptors (GPCRs) such as the hydroxy-carboxylic acid receptor 2 (HCAR2). In the mouse retina the activation of HCAR2 receptors by systemic BHB reduces ER stress and NLRP3 inflammasome associated proteins, preventing diabetic retinal damage. Interestingly, while retinal ER stress markers (pPERK, pIRE1α, ATF6) were elevated in diabetic C57BL6J mice, their levels were significantly reduced by the systemic i.p. BHB treatment twice a week for 10 weeks. Also, the pro-inflammatory cytokines (IL-1β and IL-18) were reduced by BHB treatment (Trotta et al., 2019). This results further support that attenuation of the UPR is involved in BHB protective action.

Altogether, these observations suggest that the effect of ketosis on UPR activation is context and tissue dependent. In the liver, ER stress and UPR activation, particularly the IRE1α branch, mediate the adaptive metabolic shift towards fatty acid oxidation and ketogenesis under fasting and KD conditions, while in the context of aging and disease (ischemia, low glucose and diabetes) the KD and BHB treatment down-regulate ER stress supporting proteostasis and preventing inflammation and cell damage in the liver, retina and brain. ER stress alleviation by the KD or BHB treatment involves the increase in the antioxidant enzymes, MnSOD and catalase mediated by FOXO3a, as oxidative stress can activate the UPR (Bae et al., 2016; Xie et al., 2016).

### 4.2. Ketone bodies promote the autophagy flux

Autophagy is the major bulk lysosomal degradation system to clear damaged cellular components including unfolded proteins and dysfunctional organelles. It is also involved in the recycling and turnover of long-lived proteins in order to obtain elements for the synthesis of new molecules and sustain cell homeostasis (Mizushima et al., 2008; Mizushima and Komatsu, 2011). Autophagy is particularly important in the nervous system, since as postmitotic cells, neurons have to survive for a life time. Autophagy can be initiated under different conditions, typically starvation or nutrient deprivation, but also during oxidative stress, ER calcium depletion, decreased glycosylation and protein aggregate accumulation (Mizushima et al., 2004; Gerónimo-Olvera and Massieu, 2019). Moreover, deficient autophagy can contribute to protein aggregates formation and neurodegeneration (Hara et al., 2006; Komatsu et al., 2006).

Autophagy is a conserved mechanism, first described in yeast, which involves the autophagy related proteins (ATG) (Tsukada and Ohsumi, 1993). Three different types of autophagy have been described; macroautophagy, generally referred as autophagy, where damaged molecules are sequestered in double membrane vesicles formed from the ER, known as autophagosomes, that later fuse with the lysosome for cargo degradation; microautophagy involves the recruitment of cytoplasmic sections containing damaged molecules directly into the lysosome to be degraded; and chaperon-mediated autophagy (CMA), which involves the specific degradation of substrate proteins containing a consensus sequence of five amino acids; these are recognized by chaperon hsc70 to be internalized and degraded into the lysosome, through the lysosomal membrane complex formed by hsc70 and the lysosomal receptor LAMP2A (Gerónimo-Olvera and Massieu, 2019).

Autophagosome formation initiates when kinase ULK1 is activated after mTOR inhibition during nutrient deprivation and the initiation complex, integrated by UKL1, FIP200, ATG13 and ATG101, is formed at the ER membrane. ULK1 activates class III PtdIns3K complex formed by VPS34, Beclin 1, VPS14 and ATG14 to generate PtdIns3P for the formation of the double membrane of the autophagosome. Membrane elongation is driven by two ubiquitin-like conjugation systems. The first involves the reactions between ATG12-ATG7 and ATG10 and the formation of the complex of ATG12-ATG5-ATG16, which is essential for autophagosome membrane elongation. The second system involves the conjugation of ATG8/LC3 (microtubule associated light chain 3) to phosphatidylethanolamine (PE) at the autophagosome membrane through a ubiquitin-like reaction involving ATG7 and ATG3. LC3 is transformed to its lipidated form, LC3-II, and binds to both the external and the luminal sides of the autophagosome membrane. Damaged cell components are recognized by the receptor SQSTM1/p62, which relocates cargo into the autophagosome. LC3-II is degraded with SQSTM1/p62 in the autolysosome after the fusion of the autophagosome with the lysosome. Therefore, an increase in LC3-II accompanied with a decrease in SQSTM1/p62 is taken as an index of autophagosome formation and cargo degradation, which indicates an active autophagic flux (Mizushima et al., 2008; Mizushima and Komatsu, 2011).

Several studies have suggested a link between the UPR and autophagy. Autophagy can be triggered by activation of the PERK pathway of the UPR, as several autophagic genes are downstream targets of ATF4 and CHOP, including LC3-II, SQSTM1/p62, ATG7 and ATG5 (B’Chir et al., 2013). The ER stress inductor, tunicamycin, stimulates autophagy through activation of the PERK pathway, and ATF4 depletion disrupts tunicamycin-induced autophagy (Luhr et al., 2019). On the other hand, IRE1α RNase activity and splicing of XBP1 mRNA have the opposite effect on autophagy through the downregulation of the transcription factor FOXO1 and its downstream autophagy-related genes by XBP1 (Zhou et al., 2011; Kishino et al., 2017).

Several studies support the effect of BHB on autophagy (Table 3). In human embryonic fibroblasts, BHB can stimulate CMA through the oxidation of protein substrates, facilitating their recognition by the autophagy CMA machinery and their transport to lysosomes (Finn and Dice, 2005). To date, this mechanism has not been described in the nervous system.

**Table 3:** Effects of BHB on autophagy in the nervous system.

Cell type/tissue	Treatment	Proteins affected	Outcome	Reference
Mouse spinal cord	Caloric restriction or BHB.	Decreased LC3-II, SQSTM1/p62, IL-1β, and TNFα.	Restoration of the autophagic flux and anti-inflammatory effect. Pain relief.	Liu et al. (2022)
Mouse brain	KD for 2 weeks.	Upregulation of FOXO3a, FOXO1, TFEB and SIRT2.	Increased Autophagy/mitophagy and lysosomal biogenesis	Gómora-García et al. (2023)
Rat cortical neurons	10 mM BHB from 6–48 h.	Stimulation of FOXO3a, FOXO1 and autophagy genes in a Sirt2-dependent manner. Upregulation of TFEB and PGC1α.	Increased Autophagy/mitophagy and lysosomal biogenesis. Increased resistance to glucose deprivation.	Gómora-García et al. (2023)
Rat cortical neurons	BHB 10 mM in GD and 5 mM in GR.	Decreased LC3II and SQSTM1/p62.	Increased autophagic flux and neuronal survival.	Camberos-Luna et al. (2016)
Rat brain (Epilepsy model)	KD	Increased Beclin, ATG5 and LC3II/LC3I ratio.	Stimulation of autophagy, alleviation of seizure severity and reduced number or damaged neurons.	Wang et al. (2018)
Rat brain (hypoglycemia model)	BHB (i.p: 250 mg/Kg. Two doses).	Decreased LC3II and SQSTM1/p62 in the cortex and the hippocampus.	Increased autophagic flux and reduction of the number of damaged neurons.	Torres-Esquivel et al. (2020)
Rat brain	BHB (i.v.: 250 mg + 24 h local infusion of BHB 2 M).	Decreased ATG12-ATG5, Beclin1, LC3-II, LAMP2 and SQSTM1/p62.	Attenuated autophagy activation and increased autophagic flux. Reduced lesion size.	Montiel et al. (2020)
Mouse retina	BHB (i.p: 50 and 100 mg/Kg twice a week for 10 weeks).	Restoration of BDNF and connexin-43 content; decreased LC3-II, ATG14, Beclin1, and Iba2.	Attenuation of excessive autophagy and connexin-43 degradation. Decreased microglial activation.	Trotta et al. (2022)

Ketone bodies treatment in the nervous system impact the regulation of autophagy-related proteins in different species and experimental models, changing the autophagic flux and other biological processes.

KD, Ketogenic diet. BHB, β-Hydroxybutyrate. i.p., intraperitoneal. i.v., intravenous. LC3, Microtubule-associated protein 1A/1B-light chain 3. SQSTM1/p62, Sequestosome 1 protein. IL-1β, Interleukin-1β. TNFα, Tumor necrosis factor α. FOXO, Forkhead box protein. TFEB, Transcription factor EB. SIRT2, Sirtuin 2. PGC1α, Peroxisome proliferator-activated receptor coactivator-1α. ATG, Autophagy-related protein. LAMP2, Lysosome-associated membrane glycoprotein 2. BDNF, Brain-derived neurotrophic factor.

Studies suggest that BHB stimulates the autophagic flux in several preparations. In the liver of mice fed for 4 weeks with two types of ketogenic chows composed of fat either from animal or plant origins, LC3-II increased while SQSTM1/p62 decreased, suggesting the stimulation of the autophagic flux. The signaling pathways involved mTOR inhibition and AMPK activation and were dependent on the diet composition, suggesting that plant fats exert a more profound effect on the orchestrated upregulation of autophagy (Liśkiewicz et al., 2021).

In agreement, Habieb et al. (2021) suggested an anti-aging effect of BHB treatment against hepatic senescence, an effect associated with decreased hepatic cellular damage and pro-inflammatory molecules and increased autophagic flux (Habieb et al., 2021). Conversely, Takagi et al. (2016) previously reported that autophagy is needed for ketogenesis in mice during starvation. Mice deficient in ATG5, an essential protein for autophagosome formation, show decreased lipid droplet formation and ketogenesis after 36 h starvation. Moreover, kidney ketogenesis, driven by the upregulation of HMGCS2, compensates for decreased ketone production in the liver. These observations suggest a role of autophagy in lipid metabolism and ketogenesis during starvation (Takagi et al., 2016). Together, these studies demonstrate that BHB stimulates the autophagic flux in the liver. Conversely, autophagy can regulate ketogenesis, suggesting a close link between autophagy and ketogenesis in the liver.

Several reports support that BHB stimulates autophagy in the central nervous system (Table 3). A recent study showed that 2-weeks of feeding a KD increased the content of proteins involved in autophagy and mitophagy in the brain of mice. These changes were associated with increased levels of Sirt2 and the upregulation of FOXO1, FOXO3a, and TFEB, three transcription factors involved in the regulation of autophagy, mitophagy, and lysosome biogenesis genes (Gómora-García et al., 2023). Similarly, long-term exposure (6–48 h) of cortical neurons to BHB under control culturing conditions stimulated autophagy and mitophagy through FOXO1 and FOXO3a in a Sirt2-dependent manner. The upregulation of TFEB, a master regulator of lysosomal biogenesis, was also stimulated. Moreover, BHB also increased the AMPK-dependent ULK1 phosphorylation suggesting that BHB stimulates autophagy initiation. These effects were associated with increased resistance to glucose deprivation (Gómora-García et al., 2023).

In a model of inflammatory pain in the spinal cord of mice, caloric restriction or BHB administration relieved pain and inflammation through the restoration of the impaired autophagic flux, as LC3-II and SQSTM1/p62 decreased in the spinal cord of treated mice. This effect was accompanied by a decrease in the pro-inflammatory molecules, IL-1β and TNFα (Liu et al., 2022), suggesting that the anti-inflammatory effect of D-BHB involves the stimulation of the autophagic flux.

Also, in a rat model of epilepsy, the KD attenuated neuronal injury via autophagy and mitochondrial pathways. The KD alleviated seizure severity, decreased the number of degenerating cells and raised the level of autophagic proteins and the LC3-II/LC3-I ratio, suggesting an increase in autophagy following the KD (Wang et al., 2018). Also, autophagy impairment induced by the excitotoxic lesion after NMDA administration, was rescued by the continuous local brain infusion of BHB, which also diminished the lesion size (Montiel et al., 2020).

During glucose-limiting conditions in neurons, BHB exposure prevents the accumulation of autophagosomes and increases neuronal survival by stimulating the autophagic flux (Camberos-Luna et al., 2016). Similarly, in the brain of rats subjected to severe hypoglycemia, BHB treatment restores the autophagic degradation increasing neuronal survival in the cortex and the hippocampus (Torres-Esquivel et al., 2020).

In agreement, BHB treatment protects heart from ischemia/reperfusion injury in mice. It reduced the infarct size, promoted the autophagic flux and enhanced protein expression of lysosome associated membrane protein-2 (LAMP2) in myocardium. Also, BHB treatment reduced reactive oxygen species production, enhanced ATP generation, attenuated mitochondrial swelling, and partially restored mitochondrial membrane potential in myocardium. Importantly, BHB treatment also reduced the abundance of the UPR proteins, CHOP, XBP1 and GRP78, suggesting the attenuation of the UPR (Yu et al., 2018).

Finally, a recent study suggests that increased BDNF levels and the attenuation of excessive autophagy mediate the protective effect of BHB against diabetic retinopathy. This effect is associated with the diminished degradation of connexin-43, a gap junction protein associated with retinal cell integrity. Furthermore, BHB administration significantly reduced microglial activation (Trotta et al., 2022).

Altogether, these studies suggest that in the nervous system, BHB improves autophagy through different mechanisms, which include AMPK activation, the transcriptional stimulation of autophagy and lysosomal genes, and the preservation of lysosomal function. These effects might cooperate with the maintenance of proteostasis and the regulation of the UPR.

## 5. Ketone bodies and disrupted proteostasis in Alzheimer's and Parkinson’s disease

### 5.1. Alzheimer’s disease

Aggregation of aberrant proteins into insoluble deposits is a hallmark of several neurodegenerative disorders known as proteinopathies, which include AD and PD diseases. Mutations in genes encoding amyloid beta precursor protein (APP) and presenilins 1 and 2 are characteristic of the familial type of AD and lead to the aberrant processing of APP and the production of amyloid-β peptides that aggregate into senile plaques extracellularly. Also, the hyperphosphorylation of the microtubule associated protein Tau facilitates its aggregation and formation of neurofibrillary tangles (NFTs), which are involved in synaptic disruption and neurodegeneration in Alzheimer’s disease (Lane et al., 2018).

Several studies have demonstrated the activation of the UPR in the brain of AD rodent models and patients (Chang et al., 2002; O’Connor et al., 2008; Hoozemans et al., 2009), which suggests the loss of proteostasis. Increased protein levels of GRP78 and pPERK have been observed in *postmortem* brain tissue of AD patients (Hoozemans et al., 2005), and enhanced immunoreactivity to pPERK, peIF2α, and pIRE1α in neurons correlates with Tau neuropathology (Hoozemans et al., 2009).

Persistent phosphorylation of eIF2α and protein synthesis repression causes neurodegeneration, and PERK specific inhibition reduces neuronal death in a mice model of prion disease (Moreno et al., 2012). Likewise, decreased PERK expression prevents sustained inhibition of protein translation, restores synaptic function and memory, and reduces neurodegeneration in mice models of AD (Ma et al., 2013; Devi and Ohno, 2014). Moreover, AD transgenic animals with eIF2α haploinsufficiency showed reduced amyloid-β deposition and expression of BACE1 (β-secretase called β-site APP-cleaving enzyme 1), a key secretase for the production of amyloid-β peptides and a target of ATF4 (Devi and Ohno, 2014). This observation suggests that sustained translation arrest of synaptic proteins due to persistent eIF2α phosphorylation underlies synaptic dysfunction, memory deficits, and neurodegeneration in AD. Thus, small molecules partially restoring protein translation might serve as therapeutic tools for neurodegenerative disorders (Halliday et al., 2015; Rahman et al., 2018; Hughes and Mallucci, 2019).

On the other hand, increased expression of the active form of XBP1 in the brain reduces amyloid deposits and improves memory loss. Furthermore, XBP1 administration in the hippocampus restores the content of synaptic proteins, suggesting the therapeutic potentiality of spliced XBP1 to alleviate AD neuropathology (Duran-Aniotz et al., 2023).

Defective autophagy also contributes to the accumulation of misfolded proteins and AD neuropathology, and autophagy-activating molecules might be a strategy for AD treatment (Zhang et al., 2021c). Accumulation of autophagosomes in dystrophic neurites has been reported (Yu et al., 2005), and deficient transport of autophagosomes to the soma is believed to be responsible for autophagosome accumulation, since autophagosome-lysosome fusion occurs in the soma (Nixon et al., 2005; Nixon and Yang, 2011). In addition, Beclin1, an essential protein for autophagy initiation, is reduced in the brain of AD patients, and decreased expression of Beclin1 contributes to amyloid-β deposition and neurodegeneration in an AD mice model (Pickford et al., 2008). However, other proteins of the autophagic machinery are upregulated, which suggests enhanced autophagy. Nevertheless, the autophagic flux might be defective as LC3-II and SQSTM1/p62 content increases and autolysosomes are enlarged (Bordi et al., 2016).

The potentiality of the KD and KB supplementation as therapeutic tools for the treatment of AD has been suggested (Camberos-Luna and Massieu, 2020; Lilamand et al., 2020). However, BHB exerts multiple actions and the mechanisms involved in BHB putative protective actions in AD are still not well understood. Among these mechanisms, improved mitochondrial function, ATP synthesis, mitochondrial biogenesis, the stimulation of the antioxidant defense, decreased reactive oxygen species and anti-inflammatory effects, are mainly suggested to contribute to the alleviation of AD pathology and cognitive impairment in AD mice models and patients. For further review of the subject see Rusek et al. (2019).

A pioneer study revealed that in the nematode *C. elegans* BHB supplementation extends the mean lifespan by approximately 20%, and contributes to alleviate proteotoxicity induced by β-amyloid and α-synuclein aggregation (Edwards et al., 2014). In agreement, other studies have shown that the KD reduces the content of amyloid-β peptides and pTau and improves the cognitive function in AD rodent models (Van Der Auwera et al., 2005; Kashiwaya et al., 2013). However, whether regulation of the UPR and autophagy are involved in BHB protection against AD is still an open question.

Recently, it was found that the abundance of *Hmgcs2* is associated with the autophagic clearance of APP. HMGCS2 is reduced in aged AD transgenic mice, and its overexpression in HEK293 cells stably expressing mutated APP, induced a reduction in APP and β-amyloid fragments abundance in an autophagy-dependent manner. Furthermore, *HMGCS2* overexpression stimulated ketogenesis while its silencing inhibited APP degradation, which was re-established by acetoacetate. These observations suggest that HMGCS2 improves APP degradation through the activation of autophagy mediated by KBs (Hu et al., 2017). More recently, the same group reported that HMGCS2 also regulates Tau and pTau degradation through autophagy in astrocytoma and neuroblastoma cell lines. The authors show that this effect is mediated by KBs, which promote autophagy (Hu et al., 2023). Overall, these findings support that the beneficial effects of ketogenesis in AD rodent models might be related to the stimulation of autophagy and the degradation of pathologic aggregates of amyloid-β and Tau. More investigation is needed to elucidate the relationship between improved autophagic degradation by BHB and ER stress in AD.

### 5.2. Parkinson’s disease

PD is a movement disorder associated with resting tremor, involuntary movements, bradykinesia and rigidity caused by the neurodegeneration of dopaminergic neurons in the substantia nigra. A hallmark of PD is the formation of aggregates of mutated α-synuclein, which, together with defective autophagy and mitophagy, lead to disrupted proteostasis and mitochondrial turnover (Poewe et al., 2017). Increased pPERK and peIF2α have been observed in dopaminergic neurons of the *substantia nigra* in *postmortem* brain tissue of PD patients. Moreover, increased pPERK immunoreactivity colocalizes with α-synuclein, suggesting the loss of proteostasis and the activation of the UPR in degenerating neurons (Hoozemans et al., 2007). In agreement, in a transgenic model of α-synucleinopathy, abnormal UPR activation and chronic ER stress were associated with neurodegeneration (Colla et al., 2012). Also, the enhanced expression of *Atf4* in dopaminergic neurons of the *substantia nigra* results in neurodegeneration in a model of PD (Gully et al., 2016).

Mitochondrial damage is recognized as a central factor contributing to PD neuropathology, and loss of function mutations in tensin homolog (PTEN)-induced kinase 1 (PINK1) and parkin, two proteins involved in the clearance of damaged mitochondria through mitophagy, are involved in the neuropathology of inherited PD (Kitada et al., 1998; Valente et al., 2004). Thus, mitophagy is now considered as a therapeutic target for the treatment of PD and the search for drugs enhancing mitophagy is actively increasing (Clark et al., 2021). Also, deficient degradation by the ubiquitin proteosome system (UPS), CMA, and the autophagy-lysosomal pathway have been identified in PD, which limits the degradation of α-synuclein and α-synuclein aggregates, and disrupts proteostasis (Kulkarni et al., 2023). A close interrelation between UPR and autophagy has recently been suggested for PD (Ren et al., 2021).

Several studies have shown beneficial effects of KBs in PD rodent models and PD patients (Norwitz et al., 2019). Pioneer studies by Tieu et al. (2003) showed that continuous subcutaneous infusion of BHB in a mice model of PD reduced neurodegeneration of dopamine neurons in the *substantia nigra*, preserved the levels of dopamine and dopamine metabolites, attenuated motor deficits and improved mitochondrial oxygen consumption and ATP generation (Tieu et al., 2003). Similarly, KD reduced neurodegeneration of dopaminergic neurons in the *substantia nigra* of rats treated with the dopaminergic toxin, 6-hydroxydopamine (Cheng et al., 2009). In addition, the KD improved motor and no-motor symptoms in PD patients (VanItallie et al., 2005; Phillips et al., 2018).

Many mechanisms have been suggested to underlie BHB protective effect against PD neuropathology, including improved mitochondrial metabolism, anti-inflammatory effects, antioxidant actions and increased expression of *BDNF* (Norwitz et al., 2019). However, whether downregulation of the UPR and stimulation of autophagy by BHB are involved in the enhanced survival of *substantia nigra* dopaminergic neurons in PD patients, remains to be elucidated. Considering that pathologic aggregation of α-synuclein and deficient mitophagy are two central factors in PD neuropathology, BHB seems to be a promising molecule for the treatment of this disease, as it attenuates UPR activation and stimulates autophagy/mitophagy and lysosomal biogenesis (Guo et al., 2018; Trotta et al., 2019; Gómora-García et al., 2023).

## 6. Conclusion and future directions

**Figure 3:**
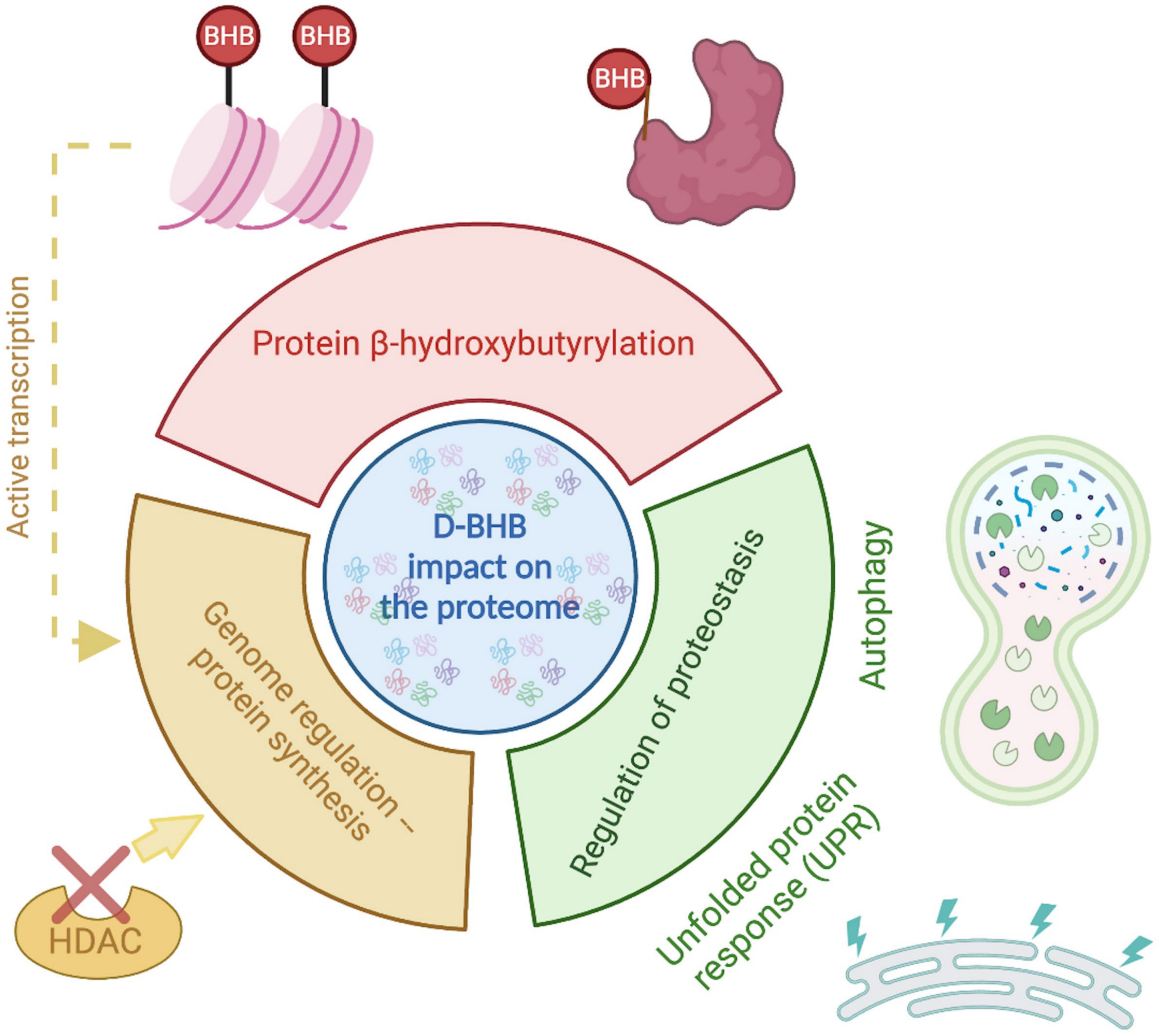
D-BHB effect on the proteome composition. The ketone body D-BHB impacts the proteome by three different mechanisms. It induces changes in the chromatin structure by the histone β-hydroxybutyrylation and the inhibition of HDACs, generating changes in the transcription that promote the enrichment of proteins mainly related to metabolic pathways. It also binds to proteins forming the PTM β-hydroxybutyrylation that may impact the protein function. And finally, it regulates the proteostasis by influencing the UPR and autophagy.

KBs are now recognized to have multifaceted functions beyond serving as an alternative energy source. BHB, the most abundant KB, confers several benefits to brain function. These beneficial effects might be partly due to its actions on proteome composition orchestrated by three main mechanisms (Figure 3). (1) the enrichment of specific pathways primarily related to metabolism; (2) β-hydroxybutyrylation of various of proteins, including histones; and (3) the regulation of proteostasis by influencing the UPR and autophagy.

The changes in proteome composition induced by increasing levels of KBs are particularly significant for tissues that heavily utilize KBs, such as the brain. BHB induces cell-type-specific changes in the CNS, including alterations in critical processes like synaptic cycle regulation, oxidative phosphorylation, and cellular metabolism in neurons.

Protein β-hydroxybutyrylation is an exciting area of research, as a wide range of proteins in various cell types and species contain Kbhb sites, sometimes multiple ones. This PTM may significantly impact protein function or even the function of protein complexes enriched in this Kbhb. Although some components of the machinery involved in the deposition and removal of Kbhb have been identified, searching for additional molecules involved in β-hydroxybutyrylation regulation is still needed. In addition, studying the distribution and abundance of β-hydroxybutyrylation using various imaging techniques is crucial to validate the *in silico* findings. Finally, more knowledge about the functional implications of the basal levels of β-hydroxybutyrylation and the changes induced by the KD or BHB exogenous administration is still needed.

Evidence supports that KBs influence the proteostasis by downregulating the UPR and enhancing the autophagic flux in the nervous system. The loss of proteostasis and impaired autophagy/mitophagy coexists in the brain of patients affected by proteinopathies like AD and PD, and these are two central factors leading to neurodegeneration. The search for molecules targeting the UPR and promoting the autophagic flux and mitophagy is a promising area of research that might lead to novel therapeutic approaches for treating neurodegenerative diseases. Studies have suggested the beneficial effects of KBs against brain proteinopathies like AD and PD; hence it is intriguing to know whether the regulation of the UPR and autophagy mediates these effects. This question remains open and demands further research. Also, additional research concerning the possible effect of Kbhb on UPR and autophagy regulation will increase our understanding of BHB actions.

## Author contributions

LG-V and LM conceived this review article, revised the literature, and critically discussed it and wrote the manuscript. All authors contributed to the article and approved the submitted version.

## Funding

LM is supported by UNAM-PAPIIT IN202922 and CONACYT A1-S-17357 grants and LG-V is supported by DGAPA-UNAM postdoctoral fellowship.

## Conflict of interest

The authors declare that the research was conducted in the absence of any commercial or financial relationships that could be construed as a potential conflict of interest.

## Publisher’s note

All claims expressed in this article are solely those of the authors and do not necessarily represent those of their affiliated organizations, or those of the publisher, the editors and the reviewers. Any product that may be evaluated in this article, or claim that may be made by its manufacturer, is not guaranteed or endorsed by the publisher.
